# Generation of iPSCs from a Patient with the M694V Mutation in the *MEFV* Gene Associated with Familial Mediterranean Fever and Their Differentiation into Macrophages

**DOI:** 10.3390/ijms25116102

**Published:** 2024-06-01

**Authors:** Elena V. Grigor’eva, Lana V. Karapetyan, Anastasia A. Malakhova, Sergey P. Medvedev, Julia M. Minina, Varduhi H. Hayrapetyan, Valentina S. Vardanyan, Suren M. Zakian, Arsen Arakelyan, Roksana Zakharyan

**Affiliations:** 1 Institute of Cytology and Genetics, Siberian Branch of Russian Academy of Sciences, 630090 Novosibirsk, Russia; evlena@bionet.nsc.ru (E.V.G.); amal@bionet.nsc.ru (A.A.M.); medvedev@bionet.nsc.ru (S.P.M.); minina_jul@bionet.nsc.ru (J.M.M.); zakian@bionet.nsc.ru (S.M.Z.); 2 Meshalkin National Medical Research Center, Ministry of Health of the Russian Federation, 630055 Novosibirsk, Russia; 3 Institute of Chemical Biology and Fundamental Medicine, Siberian Branch of Russian Academy of Sciences, 630090 Novosibirsk, Russia; 4 Department of Bioengineering, Bioinformatics, and Molecular Biology, Institute of Biomedicine and Pharmacy, Russian-Armenian (Slavonic) University, Yerevan 0051, Armenia; lana.karapetyan@rau.am (L.V.K.); varduhi.hairapetyan@rau.am (V.H.H.); arsen.arakelyan@rau.am (A.A.); 5 Institute of Molecular Biology NAS RA, Yerevan 0014, Armenia; 6 Department of Rheumatology, Yerevan State Medical University after Mkhitar Heratsi (YSMU), Yerevan 0025, Armenia; valentina.vardanyan@gmail.com; 7 Department of Rheumatology, “Mikaelyan” Institute of Surgery, Yerevan 0052, Armenia

**Keywords:** Familial Mediterranean fever, macrophages, patient-specific induced pluripotent stem cells, differentiation, *MEFV* gene

## Abstract

Familial Mediterranean fever (FMF) is a systemic autoinflammatory disorder caused by inherited mutations in the *MEFV* (Mediterranean FeVer) gene, located on chromosome 16 (16p13.3) and encoding the pyrin protein. Despite the existing data on *MEFV* mutations, the exact mechanism of their effect on the development of the pathological processes leading to the spontaneous and recurrent autoinflammatory attacks observed in FMF, remains unclear. Induced pluripotent stem cells (iPSCs) are considered an important tool to study the molecular genetic mechanisms of various diseases due to their ability to differentiate into any cell type, including macrophages, which contribute to the development of FMF. In this study, we developed iPSCs from an Armenian patient with FMF carrying the M694V, p.(Met694Val) (c.2080A>G, rs61752717) pathogenic mutation in exon 10 of the *MEFV* gene. As a result of direct differentiation, macrophages expressing CD14 and CD45 surface markers were obtained. We found that the morphology of macrophages derived from iPSCs of a patient with the *MEFV* mutation significantly differed from that of macrophages derived from iPSCs of a healthy donor carrying the wild-type *MEFV* gene.

## 1. Introduction

Familial Mediterranean fever (FMF) is a systemic autoinflammatory disorder characterized by recurrent episodes of fever and polyserositis (e.g., peritonitis, pleuritis, synovitis) symptoms. The FMF carrier frequencies are high in several eastern Mediterranean populations, ranging from 37–39% in Armenians and Iraqi Jews, to 20% in Turks, North African and Ashkenazi Jews, and Arabs, which leads to a significant economic burden [1,2]. The disease is mainly caused by recessively inherited mutations in *MEFV*, which encodes pyrin protein, which plays an important role in inflammatory processes [3]. There are two “mutation hot-spots” located in the 2nd (E148Q) and 10th (M694V, M694I, M680I, and V726A) exons. These mutations account for over 90% of all FMF cases [4]. Mutated pyrin causes an exaggerated inflammatory response by uncontrolled interleukin-1 (IL-1) secretion [5]. Besides the advances in molecular genetics of FMF, the molecular mechanisms underlying the disease are not fully understood. These questions have been studied using a battery of experimental and in silico methods. Thus, molecular dynamic simulations gain insight into the role of mutations on pyrin structure, function, and interactions [6,7]. Another study of polymorphonuclear neutrophils of FMF patients suggests increased sensitivity of mutated pyrin inflammasome towards cytoskeletal modifications in the absence of pathogens [8]. A recent study using different cell types (synovial fibroblasts, monocytes, macrophages) showed that inflammation-related functional assays have an anti-inflammatory effect of miR-197-3p [9]. Various cell-line-based models have been developed for a more comprehensive understanding of the etiology and pathogenesis of FMF [10]. Furthermore, gene editing with CRISPR/Cas9 is used to understand the effect of the *MEFV* E583A mutation on IL-1β secretion [11]. However, immortalized cell lines are limited in mimicking the disease of interest since they do not account for patient genetic variability [12], may accumulate mutations and lack genetic and cellular diversity, and mainly represent cancer-derived cells [13]. Conversely, patient primary cells have limited potential for cultivation and maintenance, posing limitations for experiments.

Induced pluripotent stem cells (iPSCs) are considered a unique tool for studying the molecular genetic mechanisms of this disease, disease modeling, and potential drug screening [14,15,16]. The main advantage of iPSCs is the almost unlimited ability of cultivation and differentiation serving as a proper source of pluripotent stem cells and any type of cells in the living organism. IPSCs were successfully used for the studying of autoinflammatory [17,18], neurodegenerative [17,18,19,20,21,22,23,24], and other diseases. There have been few attempts to create iPSCs for FMF patients, for example, a fibroblast-derived cell line of a Turkish patient with a homozygous missense mutation (p.Met694Val) in the *MEFV* gene [25].

In this study, we generated iPSCs from an Armenian FMF patient carrying a homozygous c.2080A>G (M694V) mutation in the *MEFV* gene. Molecular genetic characterization proved their stemness characteristics. We further differentiated these cells into macrophage-like cells. Morphological analysis revealed significant differences between the obtained macrophages with mutated *MEFV* gene and macrophages derived from iPSCs with wild-type *MEFV* [26].

## 2. Results

### 2.1. Generation and Characteristics of iPSCs, Associated with the MEFV Gene Mutation

**Figure 1:**
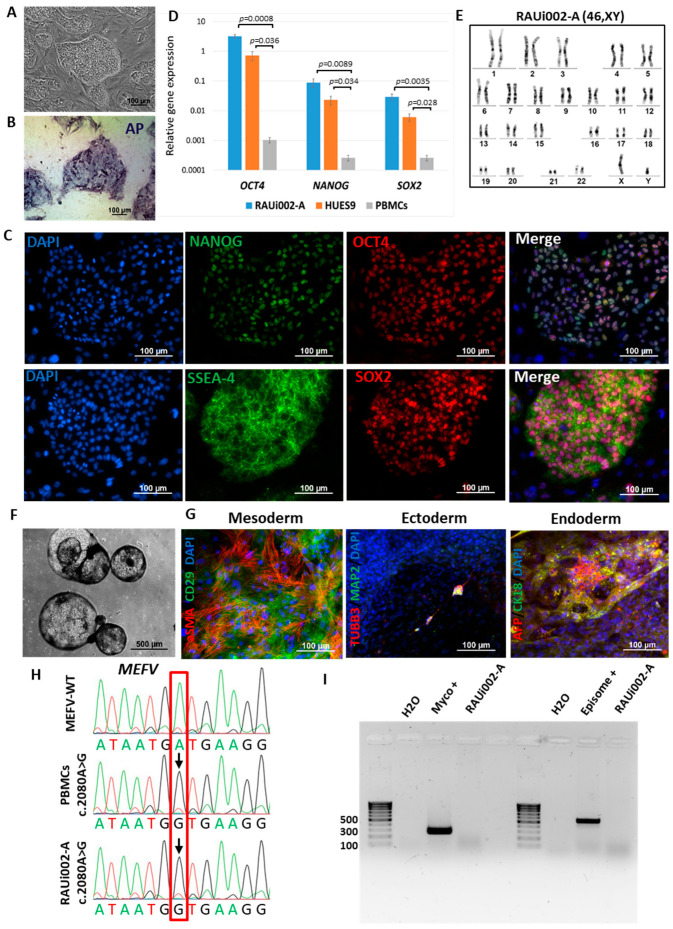
Characteristics of the iPSC cell line RAUi002-A. (**A**) Morphology of iPSC colonies. (**B**) Histochemical detection of alkaline phosphatase (AP). (**C**) Immunofluorescent staining for pluripotency markers OCT4 (red signal), NANOG (green signal), SSEA-4 (green signal), TRA-1-60 (red signal). (**D**) Quantitative analysis of the expression of pluripotency markers (OCT4, NANOG, SOX2) using RT-qPCR. Error bars indicate the standard deviation, *p*-value < 0.05, *n* = 3. Student’s t-test was used to assess statistical significance. (**E**) Karyotype analysis confirmed the presence of normal (46,XY) chromosome set. (**F**) Morphology of embryoid bodies on the 18th day of differentiation. (**G**) Immunofluorescent staining for differentiation markers: αSMA (red signal) and CD29 (green signal) (mesoderm); TUBB3/TUJ1 (red signal) and MAP2 (green signal) (ectoderm); AFP (red signal) and CK18 (green signal) (endoderm). Nuclei were stained with DAPI (blue signal). (**H**) Chromatograms of *MEFV* gene regions of PBMCs of a patient with FMF, and iPSCs with wild-type *MEFV* [26]. The position of the detected polymorphism indicated with red box. The detected polymorphism is marked with arrow. (**I**) PCR test for mycoplasma and episomes of the iPSC line (RAUi002-A). Scale bars for (**A**–**C**) and (**G**)—100 μm. Scale bar for (**F**)—500 μm.

A 20-year-old patient was admitted to the Rheumatology Department of Mikaelyan Institute of Surgery with symptoms relevant to the mixed thoracoabdominal form of FMF, including pain in joints, arthritis, erysipeloid erythema, and fever. Genetic analysis of the patient revealed a pathogenic homozygous missense mutation c.2080A>G (p.M694V, rs61752717) in exon 10 of the *MEFV* gene. We isolated peripheral blood mononuclear cells (PBMCs) in a Ficoll gradient and reprogrammed them using episomal vectors OCT4, KLF4, L-MYC, SOX2, LIN28, and Trp53 [27]. As a result, 10 independent cell lines were obtained, one of which was characterized in detail. All obtained cell lines have a large nuclear–cytoplasmic ratio, grow in densely packed iPSC-like single-layer colonies (Figure 1A), and express the early stem cell marker endogenous alkaline phosphatase (Figure 1B). Cultivation of the obtained cells was carried out on a mitotically inactivated mouse embryonic fibroblast (MEF) substrate.

One cell line was selected for detailed characterization and was registered in the Human Pluripotent Stem Cell Registry (hPSCreg, https://hpscreg.eu, accessed on 14 March 2024) under the name RAUi002-A. We carried out quantitative (RT-qPCR) and qualitative (immunofluorescence) analyses of this line for markers of pluripotent cells. Both analyses confirmed the expression of the transcription factors OCT4, SOX2, and NANOG (Figure 1C,D), as well as the expression of surface marker SSEA-4 (Figure 1C). Cytogenetic analysis (G-banding) of the obtained cells showed the presence of a normal karyotype (46,XY) (Figure 1E).

One of the main pluripotency tests is the ability of cells to give rise to all three germ layers (ectoderm, mesoderm, and endoderm). We performed spontaneous differentiation in embryoid bodies (Figure 1F) and used an immunofluorescence analysis of differentiated cells to show the expression of mesoderm markers (α-smooth muscle actin (αSMA) and the surface marker CD29), ectoderm (tubulin β 3 (TUBB3/TUJ1) and mature neural cell markers methionine aminopeptidase 2 (MAP2)), endoderm (alpha-fetoprotein (AFP), and cytokeratin 18 (CK18)) (Figure 1G). These results demonstrated that RAUi002-A cells are pluripotent and can be qualified as iPSCs.

To confirm the presence of a pathogenic mutation in the resulting iPSC line, we performed Sanger sequencing of DNA isolated from the patient’s PBMCs and RAUi002-A iPSCs and compared it to the DNA from a conditionally healthy patient. Sequencing confirmed the substitution at position 2080 A to G in exon 10 of the *MEFV* gene in both samples compared to the control DNA (Figure 1H, location of substitution indicated by arrow). In addition, to confirm the origin of the iPSCs derived from the patient’s PBMCs, we performed STR analysis of the patient’s PBMC and the RAUi002-A cells. The results showed a complete match of 25 loci from both samples (data available on request from the authors). RAUi002-A iPSCs were also analyzed for the presence/absence of residual episomes and culture contamination with mycoplasma. Both PCR analyses showed their complete absence (Figure 1I).

Taken together, these results suggest that we obtained viable iPSCs from an FMF patient that can serve as a tool to study the contribution of the p.M694V mutation in the *MEFV* gene to the pathogenesis of FMF disease.

The characteristic summary of the RAUi002-A iPSC line is shown in Table 1.

### 2.2. Generation and Characteristics of Macrophages from RAUi002-A iPSCs

**Figure 2:**
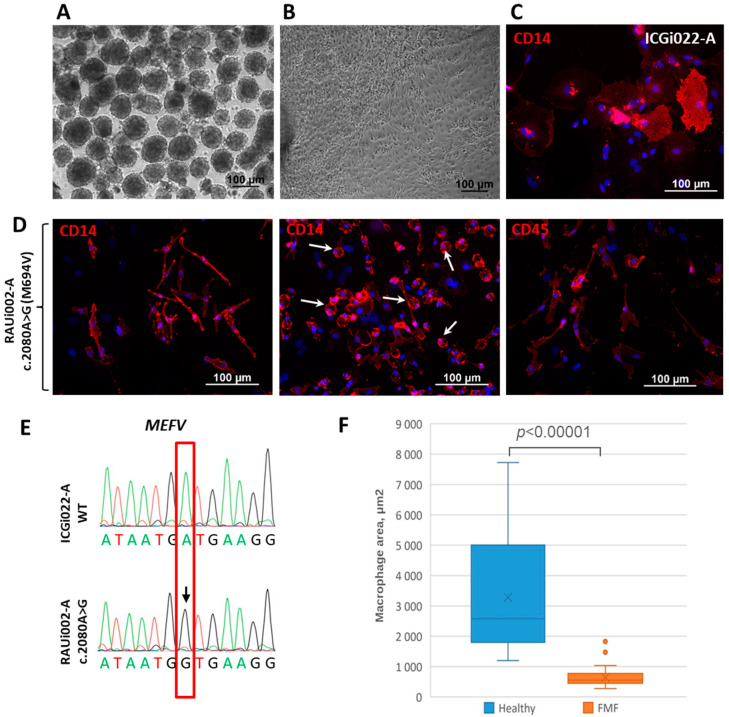
Differentiation of iPSCs into macrophages and characteristics of the resulting cells. (**A**) Morphology of embryoid bodies on the 4th day of differentiation of RAUi002-A iPSC line. (**B**) Morphology of spread out embryoid bodies on the 5th day after plating of the RAUi002-A line. (**C**) Immunofluorescence of CD14 on macrophages derived from control iPSCs line ICGi022-A. (**D**) Immunofluorescent of CD14 and CD45 on macrophages derived from RAUi002-A iPSC line. White arrows indicate rounded cells. Nuclei were stained with DAPI (blue signal). All scale bars: 100 μm. (**E**) Sanger sequencing confirmed the presence of c.2080A>G (M694V) mutation in macrophages derived from RAUi002-A iPSCs. The position of the detected polymorphism indicated with red box. The detected polymorphism is marked with arrow. (**F**) Comparison of average area of macrophages derived from FMF patient’s iPSCs and a healthy donor. *n* = 50, Mann–Whitney U test was used to assess statistical significance. *p*-value < 0.00001.

Subsequently, the RAUi002-A iPSC line was differentiated into macrophages to acquire a cell type relevant to further studies on the pathogenesis of FMF. The previously obtained iPSC line K7-4Lf/ICGi022-A was used as a control cell line in the experiments [26]. Differentiation of iPSCs into macrophages was achieved by adding the cytokines interleukin-3 (IL-3) and macrophage colony-stimulating factor (M-CSF) to the differentiating embryoid bodies (Figure 2A,B). This process led differentiation along the myeloid pathway, resulting in a homogeneous population of monocytes. Consequently, starting from the 14th day of differentiation and proceeded for over 3 weeks, monocytes were generated in the culture medium. These monocytes adhered to the plastic and, in the presence of M-CSF, terminally differentiated into cells resembling macrophages. Immunofluorescence of CD14 and CD45 mature macrophage-specific markers confirmed the identity of differentiated cells (Figure 2). The presence of the *MEFV* mutation in RAUi002-A iPSCs derivatives was again confirmed by Sanger sequencing (Figure 2E). IPSC-derived macrophages from a healthy donor were found to have a classic cloaked, spreading morphology (Figure 2C), whereas macrophages with the pathogenic p.M694V mutation in the *MEFV* gene had an elongated morphology with many rounded cells (Figure 2D, middle photo, white arrows). Similarly, we calculated the average areas of CD14-positive macrophages. The macrophages derived from FMF patient’s iPSCs had a significantly smaller area than those derived from healthy donor’s iPSCs (Figure 2F).

## 3. Discussion

In this study, we used the technology of reprogramming PBMCs into a pluripotent state to obtain patient-specific iPSCs from a patient with FMF associated with the pathogenic mutation p.M694V (according to the databases Infevers, OMIM, ClinVar, Ensembl, etc.) in the *MEFV* gene. Among the broad clinical and genetic heterogeneity of FMF, one of the most prevalent mutations substantially contributing to disease development is Met694Val (M694V). Previous studies indicated that disease severity is associated with gain-of-function mutations and, in particular, the presence of M694V homozygosity [28]. Also, it has been shown that M694V/M694V and M694V/V726A genotypes have a severe clinical course in Arab patients with FMF compared to patients with M694I/M694I genotype [29]. Moreover, it has been detected that FMF patients homozygous for M694V exhibit joint and skin-related issues, a higher rate of secondary amyloidosis, and higher colchicine dose requirements [29,30,31]. FMF patients with M694V mutation have been characterized by increased interleukin-18 (IL-18), S100A12, and caspase-1 blood levels [28]. In Turkish FMF patients, the M694V/M694V genotype has been associated with an earlier age of onset and higher frequency of arthritis and arthralgia compared with the other genotypes [30].

The cell line obtained in our study meets all the requirements of pluripotent cells, has a stem cell-like morphology, a normal karyotype, and can produce derivatives of three germ layers. These cells demonstrated their ability to differentiate into macrophages, which are one of the key cells involved in the disease pathogenesis [32].

Research related to the establishment of patient-derived iPSCs is expected to be a promising avenue for elucidating the pathogenesis of the disease, disease therapy, and drug discovery [33]. They became attractive tools for studying neurodegeneration [21,22,23,24,34], cardiac dysfunction [35,36,37], and genetic disorders, such as Duchenne’s muscular dystrophy [38]. Recently, these approaches have been actively used for modeling immune-related diseases, such as systemic lupus erythematosus, systemic sclerosis, rheumatoid arthritis, and hereditary autoinflammatory syndromes (for review, see [39]). It has been shown that various cell types differentiated from patient-derived iPSCs can be further used for research into the pathogenesis of these diseases.

To our knowledge, a few attempts have been made to generate iPSCs from FMF patients [14,25]. Fidan et al. (2015) reported a cell line derived from fibroblasts of an FMF patient carrying a homozygous p.Met694Val mutation in the *MEFV* gene [25]. In our study, we successfully reprogrammed the PBMCs of the FMF patient with a homozygous *MEFV* gene mutation (M694V). This method is less invasive for the patients. Furthermore, we differentiated stem cells into macrophages and analyzed the morphological differences between macrophages harboring mutated *MEFV* compared to those with wild-type *MEFV*. The morphology of macrophage-like cells derived from control iPSCs significantly differed from that of cells derived from iPSCs with a mutation in the *MEFV* gene. Control macrophage-like cells had a flattened morphology, whereas patient-derived cells had an elongated morphology with a large number of rounded, dying cells. We noted that under the same culture conditions, macrophages with a mutation in the *MEFV* gene are less viable, most probably due to a pathogenic mutation. These results are in concordance with the previous observations about the structural and functional features of primary immune cells of FMF patients. Thus, studies indicated characteristics of aged/activated cells (small cell size and granularity, up-regulated CXCR4) for polymorphic neutrophils from the patients in acute flares, while in remission, mixed morphology (normal cell size and granularity, up-regulated CD11b, CD49d, CXCR4, and CD62L) has been described [8].

One of the advantages of iPSC-derived macrophages is the preservation of the initial phenotype of the cells. Previous research showed that iPSC-derived cells at different stages of differentiation demonstrate a complete switch of iPSCs to cells expressing a monocyte, macrophage, or dendritic cell-specific gene profile. Moreover, iPSC-derived LPS-induced macrophages demonstrate the expression of classic macrophage pro-inflammatory response markers [40]. In addition, the ability of iPSCs to proliferate indefinitely and differentiate into various cells opens multiple avenues for studying FMF pathogenesis, screening drug candidates, and developing gene-based therapies. Using patient-specific iPSCs from FMF patients and the CRISPR/Cas9 genome editing system, it will be possible to generate modified isogenic iPSC lines with the corrected mutation, as well as introduce the mutation into control “healthy” iPSCs in the future. Thus, it will be possible to study, on isogenic lines, the contribution of this mutation to changes not only in the morphology, but also in the functional characteristics of macrophages. Such cell platforms will be valuable for understanding the effects of the mutations on pyrin inflammasome dysfunction in FMF.

## 4. Materials and Methods

### 4.1. Ethics Statement

The study was approved by the Ethics Committee of the Institute of Molecular Biology NAS RA (IRB 00004079, Protocol N3 from 23.08.2021). A patient provided informed consent about using the blood sample for planned analysis. An ICGi022-A iPSC cell line obtained from a healthy donor [26] was used as a control in the experiments of macrophage differentiation and analysis of the morphological features of mutant and wild-type *MEFV*-carrying cells.

### 4.2. Detection of the MEFV Mutation

Mutations in the *MEFV* gene in the FMF patient was determined by commercially available qPCR assay for the 26 most common mutations (FMF Multiplex real-time CPR kit, SNP Biotechnology RnD Ltd., Ankara, Turkey). This qPCR kit determines 20 mutations, which have been identified in exon 1 (E84K), in exon 2 (L110P, E148Q, E148V, E167D, E230K/Q, T267I, P283L, G304R), in exon 3 (P369S), in exon 5 (F479L), and in exon 10 (M680I (G/C-A), M694I, M694V, K695R, V726A, A744S, R761H) covering 99.2% of the mutation rate of FMF in the Anatolian, Middle East and many other countries.

### 4.3. Reprogramming of PBMCs into iPSCs

PBMCs of a patient with FMF were isolated as described previously [22]. IPSCs were obtained by overexpression of reprogramming factors OCT4, KLF4, L-MYC, SOX2, LIN28, and mp53DD using a set of episomal vectors (ID Addgene #41855–58, #41813–14) as described previously [22].

IPSCs were propagated onto the feeder layer of mitotically inactivated mouse embryonic fibroblasts (MEF) in iPSC-medium: 82% KnockOut DMEM medium, 15% KoSR, 2 mM Gluta-MAX, 100 U/mL penicillin–streptomycin, 0.1 mM MEM NEAA (all Thermo Fisher Scientific, Waltham, MA, USA), 0.1 mM β-mercaptoethanol (Sigma-Aldrich, Darmstadt, Germany), 10 ng/mL basic FGF (SCI Store, Moscow, Russia).

IPSCs were passaged using TrypLE Express (Thermo Fisher Scientific, Waltham, MA, USA), splitting 1:10 in the iPSC medium with the addition of 2 µM Thiazovivin (Sigma-Aldrich, Darmstadt, Germany) for the first 24 h.

### 4.4. In Vitro Spontaneous Differentiation of the RAUi002-A into Three Germ Layers

The differentiation capacity of the iPSCs was estimated by spontaneous differentiation in embryoid bodies, as described earlier [41].

### 4.5. Immunofluorescent Staining of the RAUi002-A iPSC Line

For immunofluorescence staining, cells growing on chambered coverglass 8-well plates (Thermo Fisher Scientific, Waltham, MA, USA) were fixed with 4% PFA (Sigma-Aldrich, Darmstadt, Germany), permeabilized with 0.5% Triton-X (Thermo Fisher Scientific, Waltham, MA, USA) in PBS for 30 min, and incubated in blocking buffer containing 1% BSA (Sigma-Aldrich, Darmstadt, Germany) in PBS at room temperature. Primary antibodies were diluted in a blocking buffer, in accordance with Table 2. Cell preparations were incubated with primary antibodies overnight at +4 °C. Preparations were washed with PBS twice for 15 min, and secondary antibodies were added for 1.5 h at room temperature. After incubation, cell preparations were washed twice with PBS and stained with DAPI. Manufacturers, catalog numbers and dilutions of all used antibodies are listed in Table 2. The preparations were analyzed using a Nikon Eclipse Ti-E (Nikon, Tokyo, Japan) microscope and NIS Elements Advanced Research version 4.30 software.

### 4.6. qPCR Analysis of Expression of Pluripotency Markers in the RAUi002-A iPSC Line

For RNA isolation, 2 × 10^6^ cells were lysed in 1 mL TRIzol reagent (Ambion by Life technologies, Carlsbad, CA, USA), and processed according to the manufacturer’s protocols. The cDNA was synthesized by reverse transcription of 1 μg RNA using M-MuLV reverse transcriptase (Biolabmix, Novosibirsk, Russia).

Quantitative PCR (qPCR) was performed on a LightCycler 480 II system (Roche, Basel, Switzerland) using BioMaster HS-qPCR SYBR Blue 2x (Biolabmix, Novosibirsk, Russia) with the following program: 95 °C, 5 min; 40 cycles: 95 °C, 10 s; 60 °C, 1 min. The primers used are listed in Table 2. The qPCR reactions for each sample were run in triplicate. CT values of the samples for *NANOG*, *OCT4,* and *SOX2* expression were normalized to actin beta (*ACTB*). Statistical analysis was performed using Student’s t-test.

### 4.7. Karyotyping of the RAUi002-A iPSC Line

Karyotype analysis was performed as described earlier [22]. For chromosome banding, samples were stained with DAPI (4,6-diamino-2-phenylindole) solution (200 ng/mL, in 2xSSC) for 5 min, then rinsed in 2xSSC buffer and water. Air-dried slides were covered with 7–10 μL antifade (Vector, Newark, CA, USA) under a coverslip. Analysis of preparations was performed using an Axioplan 2 microscope (Zeiss, Oberkochen, Germany) equipped with a CV-M300 CCD camera (JAI Corp., Yokohama, Japan) at the Center for Collective Use of Microscopic Analysis of Biological Objects at the Institute of Cytology and Genetics, Siberian Branch of the Russian Academy of Sciences. ISIS 5.0 software (MetaSystems Group, Inc., Medford, MA, USA) was used for metaphase processing and chromosome folding.

### 4.8. Genotyping of the RAUi002-A iPSC Line

Sanger sequencing was used to confirm the mutation in the *MEFV* gene in the RAUi002-A iPSC line. To verify the absence of *MEFV* mutations, Sanger sequencing was also performed for the line K7-4Lf/ICGi022-A used as a control sample. The list of primers used is shown in Table 2. Genome DNA was isolated using QuickExtract™ DNA Extraction Solution (Lucigen, Madison, WI, USA). PCR reactions were run on a T100 thermal cycler (Bio-Rad) using BioMaster HS-Taq PCR-Color (2×) (Biolabmix, Novosibirsk, Russia) with the program: 95 °C, 3 min; further 35 cycles: 95 °C, 30 s; 65 °C, 30 s; 72 °C, 30 s; and 72 °C, 5 min. For Sanger sequencing, we used BigDye Terminator V.3.1. Cycle Sequencing Kit (Applied Biosystems, Austin, TX, USA). Sequencing reactions were analyzed on an ABI 3130XL genetic analyzer at the Genomics Center of the SB RAS (http://www.niboch.nsc.ru/doku.php/corefacility, accessed on 13 March 2024).

STR profiling was performed using COrDIS Expert 26 (Moscow, Russia) by Genoanalytica (https://www.genoanalytica.ru, accessed on 14 March 2024).

### 4.9. Detection of Mycoplasma and Reprogramming Vectors in the RAUi002-A iPSC Line

The presence of episomal reprogramming vectors and mycoplasma contamination were assessed by PCR (95 °C, 5 min; 35 cycles: 95 °C, 15 s; 62 °C, 15 s; 72 °C, 20 s) using the primers listed in Table 2 [27,42]. As a positive control for episomes (Episom+, Figure 1I), mononuclear cells harvested on the 6th day after transfection were used. As a positive control of mycoplasma contamination (Mico+), we used DNA fragments of *Mycoplasma* spp. from the Myco-Visor Mycoplasma Detection Kit (Biolabmix, Novosibirsk, Russia).

### 4.10. Differentiation of the RAUi002-A iPSC Line into Macrophages

The differentiation of iPSCs into macrophages was performed according to a previously published protocol [43,44] with modifications. IPSCs were placed on a Petri dish (D60 mm) coated with mitotically inactivated MEFs. Dense iPSC colonies were detached with 0.15% collagenase type IV (Thermo Fisher Scientific, Waltham, MA, USA), washed with medium, and transferred to a Petri dish (D60 mm) coated with 1% agarose (Sigma-Aldrich, Darmstadt, Germany) in iPSC medium without the addition of bFGF. On the 4th day of culture, the formed embryoid bodies were transferred to 3 wells of a 6-well plate coated with 0.1% gelatin (Sigma-Aldrich, Darmstadt, Germany) for spreading and differentiation into monocyte-like cells in RPMI medium supplemented with 10% fetal bovine serum, 2 mM GlutaMax, 100 U/mL penicillin–streptomycin, 0.1 mM MEM NEAA, 1 mM sodium pyruvate (all Thermo Fisher Scientific, Waltham, MA, USA), 0.1 mM 2-mercaptoethanol (2-mce, Sigma-Aldrich, Darmstadt, Germany), 25 ng/mL IL-3 and 100 ng/mL M-CSF (both SCI Store, Moscow, Russia). During the 14–19 days of culture, the cell suspension was collected from embryoid bodies containing monocyte-like cells, centrifuged at 300× *g* for 5 min, and seeded onto chambered coverglass 8-well plates pretreated with 0.1% gelatin for immunofluorescence staining.

### 4.11. Calculation of Macrophage Area and Statistical Analysis

Macrophage area was calculated using ImageJ version 1.53c (NIH, Bethesda, MD, USA) software. Fifty cells positively stained for CD14 marker were analyzed for each healthy and FMF patient iPSCs-derived macrophages. The Mann–Whitney U test was performed to assess the statistical significance of the obtained results.

## Figures and Tables

**Table 1:** Characteristics and validation of the new line iPSCs RAUi002-A.

Classification	Test	Result	Data
Morphology	Photography Bright field	Normal	Figure 1A
Pluripotency status	Qualitative analysis: Alkaline phosphatase staining	Positive	Figure 1B
	Qualitative analysis: Immunocytochemistry	Positive staining for pluripotency markers: OCT3/4, SOX2, NANOG, SSEA-4	Figure 1C
	Quantitative analysis: RT-qPCR	Expression of pluripotency markers: NANOG, OCT4, SOX2	Figure 1D
Genotype	Karyotype (G-banding)	46,XY	Figure 1E
Mutation analysis	Sanger sequencing of DNA from patient’s PBMCs and iPSCs	Homozygous p.M694V (c.2080A>G, rs61752717) in exon 10 of the *MEFV* gene	Figure 1H
Differentiation potential	Embryoid body formation	Positive staining for germ layer markers: ɑSMA and CD29 (mesoderm); MAP2 and TUBB3/TUJ1 (ectoderm); CK18/AFP (endoderm)	Figure 1G
Specific pathogen-free status	Mycoplasma	Negative	Figure 1I

**Table 2:** Reagents details.

Antibodies Used for Immunocytochemistry			
	Antibody	Dilution	Company Cat # and RRID
Pluripotency Markers	Mouse IgG2b anti-OCT3/4 (C-10)	1:200	Santa Cruz Biotechnology, Dallas, TX, USA, Cat# sc-5279, RRID:AB_628051
	Mouse IgG3 anti-SSEA-4	1:200	Abcam, Cambridge, UK, Cat# ab16287, RRID:AB_778073
	Mouse IgG1 anti-NANOG	1:200	Santa Cruz Biotechnology, Dallas, TX, USA, Cat# sc-293121, RRID:AB_2665475
	Rabbit IgG anti-SOX2	1:500	Cell Signaling, Danvers, MA, USA, Cat# 3579, RRID:AB_2195767
Differentiation Markers	Mouse IgG2a anti-αSMA	1:100	Dako, Glostrup, Denmark, Cat# M0851, RRID:AB_2223500
	Mouse IgG1 anti-CD29 (Integrin beta 1) (TS2/16)	1:100	Thermo Fisher Scientific, Waltham, MA, USA, Cat # 14-0299-82, RRID:AB_1210468
	Mouse IgG2a anti-AFP	1:250	Sigma-Aldrich, Darmstadt, Germany, Cat# A8452, RRID:AB_258392
	Mouse IgG2a anti-tubulin β 3 (TUBB3)/ Clone: TUJ1	1:1000	BioLegend, San Diego, CA, USA, Cat# 801201, RRID:AB_2313773
	Chicken IgG anti MAP2	1:1000	Abcam, Cambridge, UK, Cat# ab5392, RRID:AB_2138153
	Mouse IgG1 anti-CK18	1:200	Millipore, Burlington, VT, USA Cat# MAB3234, RRID:AB_94763
Macrophage-specific Markers	Mouse IgG2b, κ anti-CD14 APC (Clone MφP9)	1:30	BD Biosciences, Franklin Lakes, NJ, USA, Cat# 345787, RRID:AB_2868813
	Mouse IgG1, κ anti-CD45 PerCP-Cy5.5 CE	1:20	BD Biosciences, Franklin Lakes, NJ, USA, Cat# 332784, RRID:AB_2868632
Secondary antibodies	Goat anti-mouse IgG3 cross-adsorbed secondary antibody, Alexa Fluor 488	1:400	Thermo Fisher Scientific, Waltham, MA, USA, Cat# A-21151, RRID:AB_2535784
	Goat anti-mouse IgG2b cross-adsorbed secondary Antibody, Alexa Fluor 568	1:400	Thermo Fisher Scientific, Waltham, MA, USA, Cat# A-21144, RRID:AB_2535780
	Goat anti-rabbit IgG (H + L) Alexa Fluor 568	1:400	Thermo Fisher Scientific, Waltham, MA, USA, Cat# A-11011, RRID:AB_143157
	Goat anti-mouse IgG1 Alexa Fluor 488	1:400	Thermo Fisher Scientific, Waltham, MA, USA, Cat# A-21121, RRID:AB_2535764
	Goat anti-mouse IgG1 Alexa Fluor 568	1:400	Thermo Fisher Scientific, Waltham, MA, USA, Cat# A21124, RRID:AB_2535766
	Goat anti-mouse IgG2a cross-adsorbed secondary antibody, Alexa Fluor 568	1:400	Thermo Fisher Scientific, Waltham, MA, USA, Cat # A-21134, RRID:AB_2535773
	Goat anti-chicken IgY (H + L) Alexa Fluor 488	1:400	Abcam, Cambridge, UK, Cat # ab150173, RRID:AB_2827653
**Primers**			
	**Target**	**Size of Band**	**Forward/Reverse Primer (5′-3′)**
Episomal plasmid vectors detection	EBNA-1	61 bp	TTCCACGAGGGTAGTGAACC/ TCGGGGGTGTTAGAGACAAC
Mycoplasma detection	16S ribosomal RNA gene	280 bp	GGGAGCAAACAGGATTAGATACCCT/ TGCACCATCTGTCACTCTGTTAACCTC
House-keeping gene (RT-qPCR)	*ACTB*	93 bp	GCACAGAGCCTCGCCTT/ GTTGTCGACGACGAGCG
Pluripotency marker (RT-qPCR)	*NANOG*	116 bp	TTTGTGGGCCTGAAGAAAACT/ AGGGCTGTCCTGAATAAGCAG
	*OCT4*	94 bp	CTTCTGCTTCAGGAGCTTGG/ GAAGGAGAAGCTGGAGCAAA
	*SOX2*	100 bp	GCTTAGCCTCGTCGATGAAC/ AACCCCAAGATGCACAACTC
Targeted mutation analysis	*MEFV*	297 bp	TGGGATCTGGCTGTCACATTG/ CATTGTTCTGGGCTCTCCGAG

## Data Availability

The data presented in this study are openly available in the Human Pluripotent Stem Cell Registry (https://hpscreg.eu/cell-line/RAUi002-A and https://hpscreg.eu/cell-line/ICGi022-A, all accessed on 13 March 2024).
